# Fluid Biomarkers for Monitoring Structural Changes in Polyneuropathies: Their Use in Clinical Practice and Trials

**DOI:** 10.1007/s13311-021-01136-0

**Published:** 2021-10-18

**Authors:** Luuk Wieske, Duncan Smyth, Michael P. Lunn, Filip Eftimov, Charlotte E. Teunissen

**Affiliations:** 1 Department of Neurology and Neurophysiology, Amsterdam Neuroscience, Amsterdam UMC, Location AMC, Amsterdam, the Netherlands; 2 Centre for Neuromuscular Disease, National Hospital for Neurology and Neurosurgery, London, UK; 3 UCL Queen Square Institute of Neurology, London, UK; 4 Neurochemistry Lab, Department of Clinical Chemistry, Amsterdam Neuroscience, Amsterdam UMC, Vrije Universiteit, Amsterdam, the Netherlands

**Keywords:** Biomarkers, Nerve damage, Polyneuropathies, Neurofilament light chain

## Abstract

Reliable and responsive tools for monitoring disease activity and treatment outcomes in patients with neuropathies are lacking. With the emergence of ultrasensitive blood bioassays, proteins released with nerve damage are potentially useful response biomarkers for many neurological disorders, including polyneuropathies. In this review, we provide an overview of the existing literature focusing on potential applications in polyneuropathy clinical care and trials. Whilst several promising candidates have been identified, no studies have investigated if any of these proteins can serve as response biomarkers of longitudinal disease activity, except for neurofilament light (NfL). For NfL, limited evidence exists supporting a role as a response biomarker in Guillain-Barré syndrome, vasculitic neuropathy, and chronic inflammatory demyelinating polyradiculoneuropathy (CIDP). Most evidence exists for NfL as a response biomarker in hereditary transthyretin-related amyloidosis (hATTR). At the present time, the role of NfL is therefore limited to a supporting clinical tool or exploratory endpoint in trials. Future developments will need to focus on the discovery of additional biomarkers for anatomically specific and other forms of nerve damage using high-throughput technologies and highly sensitive analytical platforms in adequality powered studies of appropriate design. For NfL, a better understanding of cut-off values, the relation to clinical symptoms and long-term disability as well as dynamics in serum on and off treatment is needed to further expand and proceed towards implementation.

**Supplementary Information:**

The online version contains supplementary material available at 10.1007/s13311-021-01136-0.

## Introduction

Polyneuropathies have a total prevalence of about 1% in the unselected population, and up to 7% in elderly [1]. There are many causes of polyneuropathy, the most frequent being diabetes mellitus, alcohol overuse, nutritional deficiencies, toxins and medication, genetic causes, and autoimmune and haematological disorders, whilst infections, in particular leprosy, are a more frequent cause in lower income countries. In around 20–30% of cases, the polyneuropathy has no identified cause and is idiopathic [2]. Typically, the diagnosis of a polyneuropathy is based on the clinical pattern of history and examination in combination with nerve conduction studies, while laboratory testing and other ancillary tests may be needed to clarify or confirm a potential cause that may or may not be amenable to treatment.

Arguably, the most important unmet need in the field is the monitoring of disease activity and treatment response in patients with treatable neuropathies such as chronic inflammatory demyelinating polyradiculoneuropathy (CIDP), hereditary transthyretin-related amyloidosis (hATTR), and anti-MAG neuropathy. Nerve conduction studies can be poorly tolerated by patients and are also poorly responsive to change, especially in patients with severe axonal damage. Nerve imaging with ultrasound or MRI in the twenty-first century has improved resolution, but with low specificity and inconsistent reliability when used as a response biomarker limits utility [3]. Therefore, clinical disease activity measures currently employed in clinical care and trials are mostly based on clinical outcomes such as measuring (patient reported) disability, muscle strength, and sensory deficits through clinical examination (impairments) or patient reported measures of disability. Unfortunately, a reliance solely on clinical assessment has drawbacks. In the presence of severe axonal damage (for example, in vasculitic neuropathy, severe Guillain-Barré syndrome (GBS), and paranodopathies), clinical improvement can be very delayed. In other neuropathies (for example, anti-MAG neuropathy or genetic neuropathies), progression might be too slow to capture change with clinical outcome measures, making clinical decisions difficult and clinical interventional studies very prolonged. In these patients, it would be very useful to have an early biochemical response biomarker to determine whether they are receiving optimal treatment and are likely to obtain later improvement. Finally, clinical outcomes cannot discern ongoing from residual damage and in some patients, such as those receiving maintenance intravenous immunoglobulin (IVIg) treatment for CIDP; identifying relapse or stability may avoid trial-and-error treatment withdrawal attempts, and unnecessary treatment re-initiations.

The recent development of high-sensitivity techniques to measure fluid biomarkers has accelerated biomarker research in polyneuropathies. There have recently been several reports on how biomarkers could potentially improve and accelerate diagnosis but also allow for assessment of disease activity. Most have focused on non-specific biomarkers of disease activity reflecting nerve damage. In this review, we will provide an overview of biomarker candidates that reflect structural damage across different types of neuropathies, with a focus on diagnostic and response biomarkers [4]. In particular, we will focus on neurofilament light chain (NfL) in blood as a response biomarker of disease activity in different neuropathies. Finally, we will share our view on their use in clinical care and clinical studies and future perspectives in biomarker development.

## Fluid Nerve Damage Biomarkers: What Is Known in Peripheral Nerve Disease?

At least three important challenges exist for damage biomarkers in neuropathies. Firstly, peripheral nerve tissue is of low volume and nerve-specific proteins are usually only present in very low concentrations in blood. Secondly, the tempo of damage can be acute or more importantly very prolonged, which when combined with biomarker pharmacokinetics means levels can be even lower. Therefore, assays need to have sufficient sensitivity to detect very low circulating levels. Thirdly, nerve structures, like myelin, axon, or paranode, may be preferentially or even exclusively damaged in different disorders. Therefore, it is likely that different biomarkers reflecting different nerve components are needed. While one would prefer high specificity for a diagnostic biomarker, in general response biomarkers do not need to be so specific as long as they have sufficient sensitivity and responsiveness (that is, it will closely follow changes in disease activity). Below, we have summarized biomarkers reflecting damage to different nerve structures (Table 1).

### Myelin Biomarkers

While the numerous lipids and proteins within Schwann cells and the myelin sheath are now well-characterized [5], there are currently few useful fluid biomarkers of demyelination. To date, three have shown some initial promise as biomarkers. Extracellular sphingomyelin, a widely distributed sphingolipid of the myelin sheath in peripheral and central nerves, can be measured. CSF sphingomyelin was higher in acute inflammatory demyelinating polyneuropathy (AIDP) and CIDP than in various non-inflammatory neurological disorders and axonal neuropathies including the acute motor axonal neuropathy (AMAN) variant of GBS. There was some overlap between groups, although sphingomyelin still showed relatively good sensitivity and high specificity for differentiating AIDP/active CIDP from other neurological disorders [6, 7]. Levels also correlated with clinical severity scores in both AIDP and CIDP [6]. While sphingomyelin shows promise as a diagnostic and possibly prognostic biomarker, the results need to be replicated in other studies.

Neural cell adhesion molecule (NCAM) is a member of the immunoglobulin superfamily expressed on several different neural cell types including Schwann cells. In one study, the mean serum NCAM levels were higher in demyelinating neuropathies (both inflammatory and Charcot-Marie-Tooth disease (CMT) type 1A) than in healthy controls and axonal neuropathies, and there was a positive correlation with the Overall Neuropathy Limitations Score [8]. NCAM levels were also raised, but to a lesser extent, in axonal neuropathies compared to healthy controls, and there was significant overlap between demyelinating and axonal groups, probably limiting its use as a diagnostic biomarker [8].

Serum levels of p75 neurotrophin receptor, a transmembrane protein expressed on Schwann cells and some CNS neurons, were raised in inflammatory demyelinating neuropathies but not in CMT1A, whereas NCAM levels were raised in both, indicating that there may be potential to use the levels of NCAM and p75 to differentiate CIDP from CMT in difficult cases, with a raised p75 differentiating CIDP from CMT1A with both high sensitivity and specificity [9]. The high serum levels of NCAM and p75 have been postulated to reflect increased expression by demyelinating Schwann cells and thus may not change very rapidly in acute disease.

Transmembrane protease serine 5 (TMPRSS5), a transmembrane protein expressed on Schwann cells, was identified in one study as capable of discriminating between CMT subtypes, with CMT1A patients showing increased serum levels whereas other forms of CMT did not have elevated levels [10].

### Axonal Biomarkers

The axonal cytoskeleton is made from a small number of repeating co-associating proteins, and these can be released to the extracellular space by axonal damage. By far, the best studied axonal damage markers in neurological diseases is NfL. NfL is a ubiquitous cytoskeletal protein and released into the CSF and blood in numerous CNS disorders and peripheral neuropathies (see further below). Although neurofilament heavy (NfH) was described as an axonal biomarker well before NfL and early studies of small numbers of patients showed that higher CSF levels predict axonal involvement and poor outcome in GBS [11–13], it has not been as extensively studied. Subsequent investigators described that serum NfH levels were higher in diabetic neuropathy than in diabetics without neuropathy [14], and plasma levels were higher in critical illness neuropathy/myopathy (CINM) compared to other intensive care patients [15]; once again, in these studies, there was significant overlap in NfH levels between groups. In contrast to recent NfL data, NfH was not higher in CMT compared to healthy controls [16].

The neurotrophins nerve growth factor (NGF) and brain-derived neurotrophic factor (BDNF) have been studied in diabetic neuropathy and chemotherapy-induced peripheral neuropathy (CIPN), with higher levels postulated to have a neuroprotective effect. While most studies found that lower levels of neurotrophins correlated with neuropathy and its severity [17–23], the opposite result was found in other studies [24–26], and thus, the utility of neurotrophins as biomarkers remains unclear.

Glial fibrillary acidic protein (GFAP) is an intermediate filament expressed by astrocytes in the CNS and by non-myelinating Schwann cells in the PNS. GFAP is thought to be upregulated in Schwann cells after axonal injury, and thus may be an indirect marker of axonal damage [27]. One research group found that serum GFAP was higher in axonal compared to demyelinating neuropathies; however, there was significant overlap between groups; levels were much higher in patients with multiple sclerosis than in neuropathies [27, 28]. Increased GFAP levels in CSF and serum have also been seen in GBS; however, there have been conflicting results on whether GFAP can predict long-term outcome [12, 27, 29], and whether it can differentiate AMAN from AIDP [27, 30]. Recently, serum GFAP levels were found to be higher in COVID-19-associated CINM than other critically unwell COVID-19 patients [31].

S-100B is another glial protein expressed in CNS glial cells and Schwann cells, though unlike GFAP it is found in both myelinating and non-myelinating Schwann cells [32]. S-100B levels have been found to be elevated in GBS [12, 13, 33], and levels correlated with time to recovery in one study [33] and GBS Disability Scale at 3–4 weeks in another [13]. However, levels did not predict longer term prognosis [12, 13] and could not differentiate AIDP from AMAN [13]. S-100B has not been studied in other neuropathies. As evidence on the potential to discriminate between axonal and demyelinating neuropathies is conflicting for both of these glial proteins, it remains uncertain which type(s) of structural nerve damage these biomarkers reflect.

Osteopontin is a widely expressed protein also found in Schwann cells, and is involved in inflammation and possibly axonal regeneration [34]. One study found lower serum levels at baseline that were weakly correlated with reduced sural nerve amplitude and worse clinical outcome after taxane chemotherapy; however, there was a large amount of overlap between groups [35]. In contrast, higher CSF levels of osteopontin were found in patients with GBS, with higher levels correlated with greater disability in the acute phase [36].

For disorders such as sensory neuronopathies, and infectious and inflammatory polyradiculopathies, damage to the neuronal cell body may occur at an early stage of disease. Although the axon and neuron are a continuum, and neuronal damage subsequently leads to axonal damage, some authors have advocated that neuronal proteins such as total *tau* may be used as markers for neuronal damage [37]. *Tau* and neuron-specific enolase (NSE), another neuronal protein, have both been studied in GBS, with CSF levels of *tau* associated with worse short- and medium-term outcome [12, 13], and CSF levels of NSE [33] correlated with increased time to recovery [33]. In a recent small study, there was no clear difference between plasma *tau* levels in COVID-19-positive CINM patients and other critically unwell COVID-19 patients at different timepoints [31].

In summary, for most of the molecules above, variations of study design and overlap between groups make it difficult to say whether any of them are reliable disease overarching diagnostic biomarkers (see Table 1). Most importantly, no study has focused on the potential of these molecules to serve as response biomarkers of disease activity by conducting longitudinal studies in treatable polyneuropathies. However, with the increasing availability of proteomic panels, many more potential biomarkers will likely become available in the coming years.

## Neurofilament Light Chain as a Monitoring Biomarker in Peripheral Nerve Disease

NfL is a ubiquitous axonal cytoskeletal protein present in PNS and CNS axons which forms heterodimers with the other neurofilaments alpha-internexin and peripherin [38]. Neurofilament proteins are released with other proteins into interstitial fluid during axonal damage. They can diffuse between CSF and blood, as shown by the strong correlation between levels in the serum and CSF [39]. Its potential as a CSF biomarker was shown in several CNS diseases [40]. However, since the emergence of ultrasensitive technologies enabling its detection at very low levels and in the blood, the number of neurological diseases, including neuropathies, where increased NfL is found has expanded enormously [39].

NfL is currently the best candidate as an axonal damage biomarker for a number of reasons. It is present at high level in axons, and is soluble and stable in vitro [39]. For example, NfL appears insensitive to most variations in pre-analytical handling [41]. However, there are some recovery issues with collection tubes and levels of NfL from lithium-heparin collection tubes are systematically higher than for plasma EDTA serum or citrated samples [42]. Serum and plasma NfL are both stable at room temperature for up to 7 days, and NfL is also stable to up to 4 freeze–thaw cycles and centrifugations [41, 43].

Several platforms have been developed to facilitate measurement of NfL. Serum and plasma levels are in picogrammes per millilitre in normal controls and only slightly higher in more chronic pathological processes. The majority of platforms use the antibody developed by Uman Diagnostics Measurement. Platforms vary in sensitivity (in order of decreasing sensitivity: Simoa-Ella-Siemens), their degree of automation (specialized stand-alone technologies such as Simoa and Ella vs fully clinical chemistry automates linked to robots, such as Siemens and Roche), and the platform costs. Standardization across labs and platforms is the subject of several initiatives, such as the Alzheimer’s Association Quality Control Program and Blood-Based Biomarker Working Interest Group. The development of reference material and methods is an important unmet need, to allow comparison between different platforms and assay formats and the development of unified cut-offs. There is a strong relationship between NfL levels and age, which means that cross-sectional results should always be compared against reference values obtained in the same age groups [39].

NfL levels can increase rapidly (within 12 h after hypoxic cardiac arrest [44]) and decline slowly, with an estimated half-life of 6 weeks. In vivo decline corresponding with intervention efficacy has been shown to occur within 12 weeks in multiple sclerosis patients treated with ocrelizumab [45, 46].

**Fig. 1:**
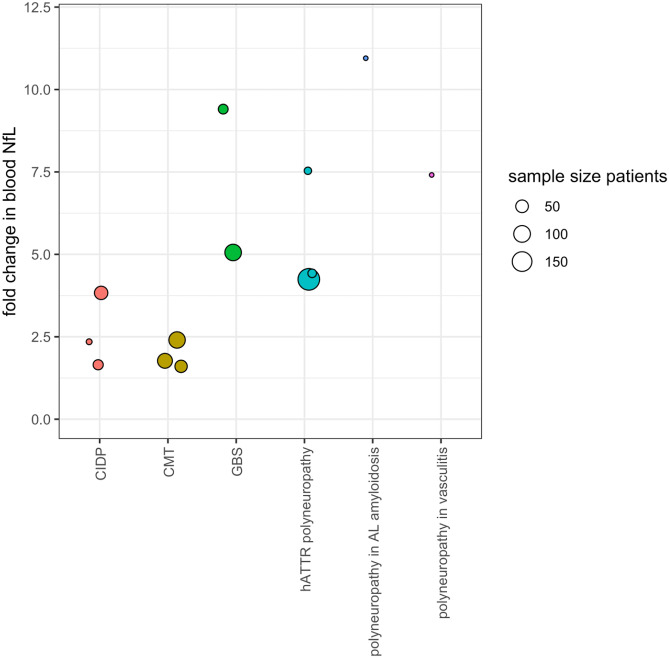
Summary of fold change in blood NfL in various polyneuropathies. This figure displays the fold change in blood NfL levels when comparing patients to healthy controls. Values are presented in Table 1. When reported, patients with active disease were chosen for this comparison. CIDP, chronic inflammatory demyelinating polyneuropathy; CMT, Charcot-Marie Tooth disease; GBS, Guillain-Barré syndrome; hATTR, hereditary transthyretin-related amyloidosis. NB two studies were excluded from this figure because they presented as outliers: Körtveleyessy [74], which reports mean NfL values, and Gaiottino [75] which uses an ECL assay

An overview of studies investigating NfL in neuropathies is provided in Table 2. Increased blood or CSF NfL levels compared to healthy controls have been found in nearly all disorders investigated. However, for many disorders, the data are limited by small numbers, unbalanced control groups (especially for age), variation in analytical platforms and methods, and limited replication. Figure 1 summarizes the relative quantitative change in NfL for various disorders compared to healthy controls. In GBS, increased blood and/or CSF NfL at the moment of diagnosis was found to predict poor outcome [29, 47]. During a 6-month course of oxaliplatin, NfL levels rose and mirrored disease severity of chemotherapy-induced polyneuropathy [48]. In CIDP, increased group NfL levels were not consistently found across studies when comparing untreated and treated patients, probably because axonal damage is not a prominent feature in all forms of CIDP [49–51]. Indeed, in neurofascin-155-mediated CIDP, NfL levels were higher compared to other forms [49]. The evidence supporting the role of NfL as a response biomarker of disease activity in neuropathies is limited. When NfL is increased in CIDP, successful treatment can lead to normalization at follow-up [49, 51]. In patients with vasculitic neuropathy, NfL reduces markedly from a peak when disease remission is achieved [52]. In a clinical trial investigating patisiran for hATTR polyneuropathy, increased NfL levels at enrolment significantly lowered during treatment while NfL levels in the placebo group continued to rise [53].

## Neurofilament Light Chain: Perspectives on Current Use in Practice and Trials

Taken together, only a handful of candidates of nerve tissue biomarkers have been explored in neuropathies. NfL has been studied most and has potential uses in clinical practice and trials. Although there is some way to go in development, we are now at an important juncture for exploring clinical applications.

### NfL in Clinical Practice

NfL is released in measurable amounts in any disorder where axonal degeneration occurs fast enough to exceed clearance. Where this occurs in a large tissue mass, released into a small volume of fluid and the axon loss is rapidly occurring, this is straightforward; for example, in prion disease and other rapidly progressive dementias, CSF NfL levels are in nanogrammes per millilitre [54]. Significantly raised levels are detectable in the blood, but usually 200-fold lower and still well-discernible from healthy controls. High levels do not necessarily correlate with other established axonal damage biomarkers (for example *tau*) and are not specific enough to be diagnostic for particular disorders, as raised levels occur in many diseases including ALS, adrenoleukodystrophy, frontotemporal dementia, and autoimmune encephalitis. In clinical scenarios where the differential diagnosis includes disorders with differing degrees of axonal degeneration, NfL may be of diagnostic help, especially early after disease onset when clinical symptoms can be limited. In a memory clinic, normal serum or CSF NfL in the ‘worried well’ can be reassuring of the lack of a neurodegenerative process [55]. Conversion to symptomatic ALS in genetic forms was shown to be preceded by a rise in NfL 12 months earlier [56]. The performance of NfL as a diagnostic biomarker in patients suspected of neuropathy has not been studied however.

As with CNS disease, there are many potential applications such as assessing treatment efficacy and prognosis, identifying relapses of relapsing and remitting diseases such as CIDP, and differentiating the worried well from those with active disease. However, NfL has not yet found a place in clinical use despite potential in GBS, CIDP, and very promising findings in hATTR [53]. Arguably, the greatest clinical utility would be in optimizing treatment of patients to identify ongoing axonal damage that might become permanent before any clinical manifestation so that effective treatment might be applied. So far, in CIDP, such a clear differentiation has not been possible [50, 51, 57]. Moreover, it remains to be shown if NfL is the best marker to identify axonal damage early, when permanent sequelae may still be preventable, or whether other damage proteins released earlier in the cascade of axonal degeneration might be better.

### NfL in Clinical Trials

Clinical trials are geared towards measuring clinically significant changes in outcome measures directly relating the therapeutic effect of an intervention to patient benefit in a reliable, reproducible, and unequivocal manner. In neuromuscular disease, measures of disability are favoured as meaningful to patients, and impairments as quantitatively measurable. However, neither relates directly to disease pathogenesis; quantifiable change may take time to develop; and as these measures are indirectly related to the pathology, they can be influenced by patient and measurement errors.

Some biological biomarkers have proven utility as outcomes in trials, such as muscle fat fraction MRI measurement in CMT1A which has excellent responsiveness and reliability in much shorter term assessments compared to clinical measures [58]. As NfL is directly linked to axonal damage, increases or decreases should be detectable acutely with worsening or with effective disease modifying interventions. This has already been shown in multiple sclerosis trials and, in polyneuropathies, only for hATTR neuropathy [53, 59].

Other biomarkers of Schwann cell or cell body damage, or perhaps immune activation, may be developed, possibly as compound measures in the future, and these may eventually become favoured for their directness. Until then, their inclusion as exploratory outcomes to prove their utility, explore responsiveness, correlate them to clinical change, and develop cut-off values will be important steps to them becoming accepted by regulatory authorities alongside traditional scales.

## Future

Now that we have the technologies and analytical platforms, as well as increasing molecular knowledge, there is huge potential for biomarkers to become significant tools in diagnosis, therapeutics, and prognosis.

For biomarker identification, peripheral nerve diseases perhaps have some advantages over CNS diseases. Firstly, the lesser tissue mass and greater volume of distribution into which NfL is released might more reliably identify severe and acute damage. And arguably, more easily quantifiable clinical outcome measures of neuromuscular disease compared to CNS pathologies may correlate better with measured levels of a blood biomarker. Changes may occur more rapidly and reliably than clinical outputs resulting in better signal to noise in outcome measurement and possibly shorter trials, at least in early phases. However, the lack of a readily available tissue correlate and limitations of disability and impairment measures in discerning ongoing or residual axonal degeneration may form challenges to accurately anchor NfL levels and define meaningful changes in polyneuropathies.

Other protein biomarkers of damage to specific cell types, which differentiate CNS from PNS disease, that quantify blood-nerve barrier dysfunction and identify different pathway perturbations need to be identified and assays developed employing the novel technologies. It is likely that compound biomarkers utilizing two or more biomarkers in panels will be useful. Reaching from single protein measures into more complex pathway biomarkers has been explored in recent novel metabolomic [60–65] and epigenetic [66–70] approaches, where these techniques have sought to identify metabolic or microRNA ‘fingerprints’ in different neuropathies. It remains to be seen whether these will be clinically useful.

Besides more biomarkers, the best fluid compartment for measurement also needs to be explored. Blood-based analysis of molecules using ultrasensitive technologies offers advantages over CSF as sampling is atraumatic and repeated samples are easily accessible giving better time resolution and more granular results. Urine and tears might also possible sources of measurable biomarkers in some cases.

Recently initiated international registries and biobanks, such as the International Guillain-Barré Syndrome Outcome Study (IGOS) [71], Inflammatory Neuropathy Consortium Base (INCbase) [72], and IgM Anti-MAG peripheral Neuropathy: from proper assessment to trial Needs (IMAGiNe) study [73], will allow collection of standardized clinical data and biomaterial of large numbers of patients that will surely speed up biomarker discovery in the next decade. Once biomarkers have been validated and rolled out for use, it will be important to unify technologies and institute quality control systems so assays remain reliable, standardized, and comparable from day to day and lab to lab. Providing core facilities for research and trials will help, but the funding for collaborative infrastructures will remain difficult to facilitate.

**Table 1:** Overview of damage biomarkers in polyneuropathies

**Biomarker**	**Study**	**Design**	**Source**	**Disease(s) studied**	**N (diseased patients)**	**Main results**
**Myelin Biomarkers**						
Sphingomyelin	Capodivento, 2017	Single centre retrospective	CSF	AIDP, CIDP	14 (total)	Sphingomyelin higher in combined AIDP + CIDP group than DC
	Capodivento, 2021	Multicentre prospective	CSF	AIDP AMAN CIDP Axonal neuropathy	12 3 53 19	Sphingomyelin higher in AIDP and CIDP than DC and axonal neuropathies; levels correlated with clinical severity scores
Neural cell adhesion molecule (NCAM)	Niezgoda, 2017	Single centre prospective	Serum	AIDP CIDP MMN Axonal neuropathy with raised CSF protein Diabetic neuropathy	40 29 11 40 20	NCAM higher in AIDP, CIDP and MMN than axonal neuropathies and HC; levels correlated with ONLS in demyelinating neuropathies
	Kim, 2019	Single centre prospective	Serum	AIDP AMAN CIDP CMT1A	14 20 36 39	NCAM higher in AIDP, CIDP and CMT1A than HC; non-significantly higher in AMAN than HC
P75 neurotrophin receptor	Kim, 2019	Single centre prospective	Serum	AIDP AMAN CIDP CMT1A	14 20 36 39	p75 higher in AIDP and CIDP than HC; non-significantly higher in AMAN than HC; in CMT1A levels were similar to HC
Transmembrane protease serine 5	Wang, 2020	Multicentre prospective	Plasma	CMT	47	TMPRSS5 higher in CMT1A, not in other forms of CMT
**Axonal Biomarkers**						
Neurofilament heavy chain (NfH)	Petzold, 2006	Single centre prospective	CSF	GBS (74% with axonal degeneration)	23	High NfH correlated with axonal involvement and poor outcome
	Petzold, 2009	Multicentre prospective	CSF	GBS	38	Mean NfH not higher in GBS compared to DC; however, high levels correlated with poor outcome (Hughes score ≥3); levels higher in poor outcome GBS than CIDP
	Wang, 2013	Single centre prospective	Serum and CSF	AIDP AMAN	11 11	Serum and CSF levels higher in AIDP and AMAN than controls (‘benign headache’); higher CSF levels correlated with GDS in AMAN acutely, at 3-4 weeks and at 13-14 weeks
	Qiao, 2015	Single centre prospective	Serum	Diabetic neuropathy	23	NfH higher in NCS-confirmed diabetic neuropathy than T2DM without neuropathy
	Wieske, 2014	Single centre prospective	Plasma	CINM	18	Peak NfH levels higher in CINM than other ICU patients
	Rossor, 2016	Single centre prospective	Serum	CMT	90	No significant difference in NfH between CMT and HC; no significant difference in levels over 1 year in CMT cohort
Neurofilament light chain (NfL)	Louwsma, 2021	Single centre retrospective	Serum	polyneuropathy in AL amyloidosis	10	See table 2 for further details on NfL studies
	Louwsma, 2021	Single centre retrospective	Serum	hATTR polyneuropathy	15	
	Ticau, 2021	Multicentre prospective	Plasma	hATTR polyneuropathy	189	
	Maia, 2020	Multicentre retrospective	Plasma	hATTR polyneuropathy	26	
	Kapoor, 2019	Single centre prospective	Plasma	hATTR polyneuropathy	20	
	Millere, 2021	Single centre prospective	Plasma	CMT	96	
	Sandelius, 2018	Single centre prospective	Plasma	CMT	75	
	Wang, 2020	Multicentre prospective	Plasma	CMT	47	
	Bischof, 2017	Single centre retrospective	Serum	polyneuropathy in vasculitis	10	
	Frithiof, 2021	Single centre prospective	Plasma	CINM	11	
	Kim, 2020	Single centre prospective	Serum	CIPN	24	
	Mariotto, 2018	Single centre prospective	Serum	GBS	5	
	Altmann, 2020	Single centre retrospective	Serum	GBS	27	
	Martín-Aguilar, 2020	Multicentre prospective	Serum and CSF	GBS	98 24	
	Körtveleyessy, 2020	Single centre retrospective	Serum and CSF	GBS	21 21	
	Gaiottino, 2013	Single centre retrospective	Serum and CSF	GBS	20 20	
	Axelsson, 2018	Single centre retrospective	CSF	GBS	18	
	Mariotto, 2018	Single centre prospective	Serum	Multifocal motor neuropathy	3	
	Mariotto, 2018	Single centre prospective	Serum	CIDP	12	
	Fukami, 2021	Multicentre prospective	Serum	CIDP	58	
	Godelaine, 2021	Single centre retrospective	Serum	CIDP	76	
	Hayashi, 2021	Single centre retrospective	Serum	CIDP	11	
	van Lieverloo, 2019	Multicentre prospective	Serum	CIDP	80	
	Mariotto, 2018	Single centre prospective	Serum	Anti-MAG polyneuropathy	3	
Nerve growth factor (NGF)	Sun, 2018	Single centre prospective	Serum	Diabetic neuropathy	65	NGF lower in diabetic neuropathy than T2DM without neuropathy and HC; levels correlated with various measures of diabetes severity
	Farajdi, 1990	Single centre prospective	Serum	Diabetic neuropathy	18	NGF lower in diabetic neuropathy than HC; lower levels correlated with lower motor conduction velocity
	Kim, 2009	Multicentre cross-sectional	Serum	Diabetic neuropathy	89	NGF higher in diabetic neuropathy than T2DM without neuropathy; however, lower levels correlated with increasing disability score
	Cavaletti, 2004	Multicentre prospective	Plasma	CIPN (cisplatin + paclitaxel)	34	Decrease in NGF correlated with increasing neuropathy severity (TNS); levels did not predict long-term outcome
	Youk, 2017	Single centre prospective	Serum	CIPN (bortezomib, vincristine, thalidomide)	45	NGF decreased after chemotherapy in patients with CIPN but did not change in patients who did not develop CIPN
	De Santis, 2000	Single centre prospective	Serum	CIPN (various agents)	23	NGF decreased after chemotherapy; lower levels correlated with severity of neuropathy
	Velasco, 2017	Single centre prospective	Serum	CIPN (various agents)	48	NGF increased after chemotherapy in patients developing painful CIPN; higher levels correlated with neuropathy severity
Brain derived neurotrophic factor (BDNF)	Sun, 2018	Single centre prospective	Serum	Diabetic neuropathy	65	BDNF lower in diabetic neuropathy than T2DM without neuropathy and HC; levels correlated with various measures of diabetes severity
	Azoulay, 2014	Single centre prospective	Plasma	CIPN (bortezomib)	25	BDNF decreased after chemotherapy in patients developing CIPN but not in patients who did not develop neuropathy
	Azoulay, 2019	Single centre prospective	Serum	CIPN (bortezomib, vincristine)	45	Low baseline BDNF correlated with development of CIPN
	Szudy-Szczyrek, 2020	Single centre prospective	Serum	CIPN (bortezomib, thalidomide)	91	High baseline BDNF correlated with severity of neuropathy (CT-CAE)
Glial fibrillary acidic protein (GFAP)	Notturno, 2008	Single centre prospective	Serum and CSF	AIDP AMAN CIDP	20 17 20	Serum GFAP higher in AMAN, AIDP and CIDP than HC; serum and CSF GFAP higher in AMAN than AIDP; levels correlated with Hughes score at 6 months
	Notturno, 2009	Single centre prospective	Serum	CIDP MMN Axonal neuropathies PMA	30 26 30 15	GFAP higher in axonal neuropathies than CIDP, MMN and HC; levels correlated weakly with ONLS
	Petzold, 2009	Multicentre prospective	CSF	GBS	38	GFAP higher in GBS than DC; no correlation between GFAP and poor outcome (Hughes score ≥3)
	Axelsson, 2018	Single centre retrospective	CSF	GBS	18	GFAP higher in GBS than healthy controls; levels at onset higher in patients with poor outcome (Hughes score ≥3)
	Frithiof, 2021	Single centre prospective	Plasma	CINM (SARS-CoV-2 positive)	11	GFAP higher in SARS-CoV-2 +ve CINM compared with other SARS-CoV-2 +ve ICU patients without CINM
S-100B	Mokuno, 1994	Single centre prospective	CSF	GBS	24	S-100B raised in 46% of GBS patients; higher levels correlated with time to recovery
	Wang, 2013	Single centre prospective	Serum and CSF	AIDP AMAN	11 11	Serum and CSF levels higher in AIDP and AMAN than controls (‘benign headache’); higher CSF levels correlated with GDS in AIDP acutely and at 3-4 weeks; no correlation at 13-14 weeks
	Petzold, 2009	Multicentre prospective	CSF	GBS	38	S-100B higher in GBS compared to DC; no correlation between levels and poor outcome (Hughes score ≥3)
Osteopontin	Han, 2014	Single centre prospective	Plasma and CSF	AIDP AMAN	24 27	CSF but not serum levels higher in AIDP and AMAN than DC (non-inflammatory neurological conditions); higher CSF levels correlated with peak GDS in the acute phase
	Pizzamiglio, 2020	Single centre prospective	Serum	CIPN (paclitaxel, docetaxel)	50	Lower baseline levels correlated with reduction in sural SNAP amplitude and poor/intermediate outcome (TNS-reduced)
**Neuronal markers**						
*Tau*	Wang, 2013	Single centre prospective	Serum and CSF	AIDP AMAN	11 11	Serum and CSF levels higher in AIDP and AMAN compared to controls (‘benign headache’); higher CSF levels correlated with GDS in AMAN acutely and at 3-4 weeks; no correlation at 13-14 weeks
	Petzold, 2009	Multicentre prospective	CSF	GBS	38	Tau not different in GBS and DC; higher levels in patients with Hughes F-score ≥2
	Frithiof, 2021	Single centre prospective	Plasma	CINM (SARS-CoV-2 positive)	11	Levels non-significantly higher in SARS-CoV-2 +ve CINM than other SARS-CoV-2 +ve ICU patients
Neuron specific enolase (NSE)	Mokuno, 1994	Single centre prospective	CSF	GBS	24	NSE raised in 42% of GBS patients; higher levels correlated with time to recovery

*AIDP*, acute inflammatory demyelinating polyradiculoneuropathy variant of Guillain-Barré syndrome; *CIDP*, chronic inflammatory demyelinating polyradiculoneuropathy; *DC*, diseased controls; *AMAN*, acute motor axonal neuropathy variant of Guillain-Barré syndrome; *MMN*, multifocal motor neuropathy; *HC*, healthy controls; *ONLS*, Overall Neuropathy Limitations Score; *CMT*, Charcot-Marie-Tooth disease; *GBS*, Guillain-Barré syndrome; *GDS*, GBS Disability Scale; *NCS*, nerve conduction studies; *T2DM*, type 2 diabetes mellitus; *CINM*, critical illness neuropathy/myopathy; *ICU*, intensive care unit; *CIPN*, chemotherapy-induced peripheral neuropathy; *TNS*, total neuropathy score; *CT-CAE*, Common Terminology Criteria for Adverse Events; *PMA*, primary muscular atrophy; *SARS-CoV-2*, severe acute respiratory syndrome-coronavirus-2 infection; *SNAP*, sensory nerve action potential

**Table 2:** Overview of neurofilament light chain in polyneuropathies. This shows the different studies that have investigated neurofilament light chain (NfL) in blood or cerebrospinal fluid in polyneuropathies. Italicized entries indicate significance

Disorder	Study	Design	Assay	Source	Cross-sectional comparisons		Longitudinal studies	
					Groups	Fold change	Groups/intervention	Conclusion
Polyneuropathy in AL amyloidosis	Louwsma, 2021	Single centre Retrospective	Simoa	Serum	AL/PNP + (*N*: 10) vs HC (*N*: 10)	*11 (149 vs 13.6)*	Not performed	Not performed
					AL/PNP* − *(*N*: 10) vs HC (*N*: 10)	*1.7 (22.7 vs 13.6)*		
					AL/PNP + (*N*: 10) vs AL/PNP* − *(*N*: 10)	*6.6 (149 vs 22.7)*		
hATTR polyneuropathy	Louwsma, 2021	Single centre Retrospective	Simoa	Serum	hATTR/PNP + (*N*: 15) vs HC (*N*: 15)	*7.5 (66.4 vs 8.8)*	Not performed	Not performed
					hATTR carriers (*N*: 15) vs HC (*N*: 15)	0.8 (6.9 vs 8.8)		
					hATTR/PNP + (*N*: 15) vs hATTR carriers (*N*: 15)	*9.6 (66.4 vs 6.9)*		
					hATTR/PNP + PND > 1 (*N*: 7) vs hATTR/PNP + PND1 (*N*: 8)	*Higher (details NR)*		
	Ticau, 2021	Multicentre Prospective	Simoa	Plasma	hATTR/PNP + (*N*: 189) vs HC (*N*: 57)	*4.3 (69.4 vs 16.3)*	hATTR/PNP + treated with patisiran (*N*: 136) or placebo (*N*: 53)	Lower at 18 months (fold change ~ 0.5)
	Maia, 2020	Multicentre Retrospective	Simoa	Plasma	hATTR carriers (*N*: 16) vs HC (*N*: 16)	Similar (details NR)	Not performed	Not performed
					hATTR/PNP + (*N*: 26) vs hATTR carriers (*N*: 16)	*4.8–15.4 (PND 1 or* > *1)*		
	Kapoor, 2019	Single centre Prospective	Simoa	Plasma	hATTR/PNP + (*N*: 20) vs HC (*N*: 16)	*4.4 (68.4 vs 15.5)*	Not performed	Not performed
CMT	Millere, 2021	Single centre Prospective	Simoa	Plasma	CMT (*N*: 96) vs HC (*N*: 60)	*2.4 (12.5 vs 5.2)*	Not performed	Not performed
					CMT1X (*N*: 10) vs other CMT (*N*: 86)	*1.3 (16 vs 12.5)*		
	Sandelius, 2018	Single centre Prospective	Simoa	Plasma	CMT (*N*: 75) vs HC (*N*: 67)	*1.8 (26 vs 14.6)*	Stable CMT (*N*: 9), HC (*N*: 13), no intervention	Unchanged at 1 year
					Severe CMT (*N*: 14) vs milder CMT (*N*: 61)	*Higher (details NR)*		
	Wang, 2020	Multicentre Prospective	PEA	Plasma	CMT (*N*: 47) vs HC (*N*: 41)	*1.6 (details NR)*		
Polyneuropathy in vasculitis	Bischof, 2017	Single centre Retrospective	Simoa	Serum	Vasculitis + PNP (*N*: 10) vs HC (*N*: 30)	*7.4 (215 vs 29)*	Active disease vs remission (*N*: 10), various treatments	Lower at 13 months (fold change 0.27)
					Vasculitis + PNP (*N*: 10) vs vasculitis − PNP (*N*: 10)	*5 (215 vs 43)*		
CINM	Frithiof, 2021	Single centre Prospective	Simoa	Plasma	ICU + CINM (*N*: 11) vs ICU − CINM (*N*: 7)	*Higher (details NR)*	Not performed	Not performed
Chemotherapy-induced polyneuropathy	Kim, 2020	Single centre Prospective	Simoa	Serum	CIPN grade 2 (*N*: 19) vs CIPN grade 0–1 (*N*: 10)	1.4 (127 vs 91.6)	Before, during, and after oxaliplatin treatment (*N*: 34)	Higher at 3 months vs baseline (fold change 1.8) and at 6 months vs 3 months (fold change 5.2); lower after stopping
					CIPN grade 3 (*N*: 5) vs CIPN grade 0–1 (*N*: 10)	*4 (373 vs 91.6)*		
Guillain-Barré syndrome	Mariotto, 2018	Single centre Prospective	Simoa	Serum	GBS (*N*: 5) vs HC (*N*: 25)	*Higher (details NR)*	Not performed	Not performed
	Altmann, 2020	Single centre Retrospective	Simoa	Serum	GBS (*N*: 27) vs HC (*N*: 22)	*9.4 (85.5 vs 9.1)*	Not performed	Not performed
	Martín-Aguilar, 2020	Multicentre Prospective	Simoa	Serum	GBS (*N*: 98) vs HC (*N*: 53)	*5 (40 vs 7.9)*	GBS (*N*: 33)	Normalized at 1 year; higher levels predict poor outcome
				CSF	GBS (*N*: 24) vs HC (*N*: 10)	*1.8 (883.6 vs 493.8)*		
	Körtveleyessy, 2020	Single centre Retrospective	Simoa	Serum	GBS (*N*: 21) vs HC (*N*: 19)	*51.3 (2603 vs 50.7)*	Not performed	Not performed
				CSF	GBS (*N*: 21) vs HC (*N*: 19)	*6.8 (7623 vs 1114)*		
	Gaiottino, 2013	Single centre Retrospective	ECL	Serum	GBS (*N*: 20) vs HC (*N*: 67)	*Serum 24 (79.4 vs 3.3)*	Not performed	Not performed
				CSF	GBS (*N*: 20) vs NC (*N*: 67)	*CSF 4.2 (1361 vs 324)*		
	Axelsson, 2018	Single centre Retrospective	ELISA	CSF	GBS (*N*: 18) vs HC (*N*: 28)	*5 (1147 vs 228)*	GBS (*N*: 18; *N*: 3 with poor outcome at long term)	Higher levels at onset in GBS with poor outcome at long term (fold change 130)
Multifocal motor neuropathy	Mariotto, 2018	Single centre Prospective	Simoa	Serum	MMN (*N*: 3) vs HC (*N*: 25)	Similar (details NR)	Not performed	Not performed
CIDP	Mariotto, 2018	Single centre Prospective	Simoa	Serum	CIDP (*N*: 12) vs HC (*N*: 25)	*Higher (details NR)*	Not performed	Not performed
	Fukami, 2021	Multicentre Prospective	Simoa	Serum	CIDP (*N*: 58) vs HC (*N*: 14)	*3.8 (29.6 vs 7.7)*	NF-155 + (*N*: 8), various treatments	Lower at 11 months (details not reported)
					Tx − (*N*: NR) vs Tx + (*N*: NR)	Similar (32 vs 28.3)		
					NF-155 + (*N*: 13) vs NF-155* − *(*N*: 45)	*2.1 (46.7 vs 22.3)*		
	Godelaine, 2021	Single centre Retrospective	ECL	Serum	Not performed	Not performed	CIDP (*N*: 76; median NfL 28.3), various treatments	Higher levels at baseline associated with progression and non-responder at 1 year
	Hayashi, 2021	Single centre Retrospective	Simoa	Serum	Tx* − *(*N*: 11) vs HC (*N*: 7)	*2.3 (23.6 vs 10.1)*	Not performed	Not performed
					Tx* − *(*N*: 11) vs remission (*N*:9)	*1.9 (23.6 vs 12.4)*		
	van Lieverloo, 2019	Multicentre Prospective	Simoa	Serum	Tx* − *(*N*: 29) vs HC (*N*: 30)	*1.6 (41.9 vs 25.5)*	Tx* − *(29), after various treatments	Similar in responders and non-responders at 6 months, normalized if elevated
					Active (*N*: 24) vs stable (*N*: 29)	*1.1 (35.8 vs 31.3)*	Not applicable	Not applicable
					Tx + (*N*: 24) vs HC (*N*: 30)	Similar (27.2 vs 25.5)	Tx + (24), IVIg withdrawal	Similar in relapsers and non-relapsers at 6 months, if elevated relapse
					Remission (*N*: 27) vs HC (*N*: 30)	Similar (29.6 vs 25.5)	Not applicable	Not applicable
Anti-MAG polyneuropathy	Mariotto, 2018	Single centre Prospective	Simoa	Serum	Anti-MAG (*N*: 3) vs HC (*N*: 25)	*Higher (details NR)*	Not performed	Not performed

*Simoa*, single molecule array; *PEA*, proximity extension assay as performed with Olink; *PNP*, polyneuropathy; *HC*, healthy controls; *hATTR*, hereditary transthyretin-related amyloidosis; *PND*, Polyneuropathy Disability Score; *CMT*, Charcot-Marie-Tooth; *CINM*, critical illness neuropathy/myopathy; *ICU*, intensive care unit; *CIPN*, chemotherapy-induced polyneuropathy; *GBS*, Guillain-Barré syndrome; *ECL*, electrochemiluminescence; *ELISA*, enzyme-linked immunosorbent assay; *DC*, neurological control; *CSF*, cerebrospinal fluid; *MMN*, multifocal motor neuropathy; *NF-155*, neurofascin-155; *Tx − *, treatment-naïve; *Tx* + , on treatment

## Supplementary Information

Below is the link to the electronic supplementary material.

Supplementary file1 (PDF 595 KB)

Supplementary file2 (PDF 356 KB)

Supplementary file3 (PDF 373 KB)

Supplementary file4 (PDF 356 KB)
